# Noncanonical function of the *Sexlethal* gene controls the protogyny phenotype in *Drosophila melanogaster*

**DOI:** 10.1038/s41598-022-05147-5

**Published:** 2022-01-27

**Authors:** Ki-Hyeon Seong, Siu Kang

**Affiliations:** 1 RIKEN Cluster for Pioneering Research, RIKEN Tsukuba Institute, Tsukuba, Ibaraki Japan; 2 Graduate School of Science and Engineering, Yamagata University, Jonan, Yonezawa, Yamagata Japan; 3 AMED-CREST, AMED, Chiyoda-ku, Tokyo, Japan; 4 Present Address: Institute of Agrobiological Sciences, NARO, Tsukuba, Ibaraki Japan

**Keywords:** Developmental biology, Genetics

## Abstract

*Drosophila melanogaster* females eclose on average 4 h faster than males owing to sexual differences in the pupal period, referred to as the protogyny phenotype. Here, to elucidate the mechanism underlying the protogyny phenotype, we used our newly developed *Drosophila* Individual Activity Monitoring and Detecting System (DIAMonDS) that detects the precise timing of both pupariation and eclosion in individual flies. Although sex transformation induced by *tra-2*, *tra* alteration, or *msl-2* knockdown-mediated disruption of dosage compensation showed no effect on the protogyny phenotype, stage-specific whole-body knockdown and mutation of the *Drosophila* master sex switch gene, *Sxl*, was found to disrupt the protogyny phenotype. Thus, *Sxl* establishes the protogyny phenotype through a noncanonical pathway in *D. melanogaster.*

## Introduction

The time taken to reach sexual maturity is often unequal between the sexes of numerous animal species. Protogyny refers to the phenotype characterized by females maturing first, whereas protandry refers to the phenotype characterized by males maturing earlier than females^1,2^. Information within the AnAge database (https://genomics.senescence.info/species/) reveals that approximately one-third of the animals across the animal kingdom show sexual dimorphism in the sexual maturation timing, with poikilotherms and homeotherms tending to exhibit protandry and protogyny, respectively (Supplementary Table S1)^3^. In insects, the terms protandry and protogyny refer to male- and female-specific differences, respectively, in the timing of adult emergence^4^. Although the AnAge database includes minimal data on arthropods, several reports have indicated that male adults tend to emerge earlier than females in many insect species^2,5–8^. Several hypotheses have been proposed to explain the occurrence of protogyny and protandry with respect to increasing fitness. There are some evolutional explanations for sexual maturation based on direct and indirect effects. Some studies have suggested that biased sexual maturation is generally a by-product of sexual size dimorphism (SSD)^2,7^. On the other hand, some reports suggested that the difference in sexual maturation timing of both sexes has fitness consequences and that selection directly maintains biased sexual maturation phenotype^2,8,9^. However, the detailed mechanism and a convincing generalized evolutional explanation for the biased sexual maturation trait remain to be provided^1,4,7,8,10,11^.

To understand the evolutionary significance of the sex bias in the sexual maturation time point, it is also important to elucidate the molecular mechanism underlying the protogyny and protandry phenotypes. However, these molecular aspects remain unclear, mainly owing to difficulties in precisely measuring the timing of maturation of individuals simultaneously and for a long period with the currently available techniques.

In the fruit fly (*Drosophila melanogaster*), adult females emerge quickly, before males (protogyny phenotype), with only a 4-h difference in eclosion timing^12^. Therefore, *D. melanogaster* offers a potentially useful model to elucidate the molecular mechanism underlying the sexual dimorphism in sexual maturation. We established a new system, *Drosophila* Individual Activity Monitoring and Detection System (DIAMonDS), which can automatically detect the phase-conversion timing of individual flies, such as the timing of pupariation, adult eclosion, and death, with high temporal resolution^13^. DIAMonDS enables time-lapse- and multi-scanning to simultaneously determine the time points of pupariation and eclosion of individuals under several chemical and environmental conditions and against different genetic backgrounds. As DIAMonDS acquires time-lapse images using a basic flatbed charge-coupled device (CCD) scanner, flies continue to catch the light signal intermittently throughout the day during the time-lapse scanning by DIAMonDS. Therefore, DIAMonDS eliminates the influence of the light-dark cycle on eclosion. Using DIAMonDS, we can precisely detect the 4-h sex-specific difference in eclosion timing, which was found to be solely due to a difference in pupal duration^13^. Because previous reports also corroborate this result, the 4-h difference in adult emergence between sex seems to reflect intrinsic developmental time to eclosion without the effect of light conditions^12^.

In *D. melanogaster*, the sex is determined by the master switch *Sex lethal* (*Sxl*) gene, which encodes an RNA splicing enzyme, by modulating the ratio of sex chromosomes to autosomes (X:A)^14,15^. In females, *Sxl* activates the *transformer* (*tra*) gene by correct splicing, while functional Tra regulates the splicing of the *doublesex* (*dsx*) gene with transformer 2 (Tra-2) as a cofactor to produce Dsx^F^, the female-specific Dsx. In contrast, males have no functional Tra protein and express male-specific Dsx^M^. Hence, these Dsx^F^ and Dsx^M^ proteins induce sex-specific phenotypic changes^16–18^, and the presence or absence of Tra or Tra-2 plays a critical role in determining sexual differentiation. Sex chromosome dosage compensation is differentially regulated by sex, and the male-specific lethal (MSL) complex is a key player in the dosage compensation machinery in *Drosophila*^19,20^. In females, dosage compensation is blocked by *Sxl*-dependent repression of *msl-2*, which encodes a limiting subunit of the MSL complex^21^. Although several studies indicate that the genes involved in the sex determinant pathway play a significant role in the expression of the sexual dimorphic traits, its contribution per se for determining the protogyny phenotype has not been reported to date^22,23^.

In this study, we applied DIAMonDS to evaluate the genetic regulation of the protogyny phenotype in *D. melanogaster*. As fruit flies alter their developmental rates when exposed to different environmental conditions^24–26^, we first explored the effect of temperature and nutrients on the protogyny phenotype. Next, we manipulated *tra* and *tra-2* to change the sex of the flies and evaluated the effect on the protogyny phenotype. Since sex chromosome dosage compensation is differentially regulated by sex, and the male-specific lethal complex plays a key role in the dosage compensation machinery in *Drosophila*^19,20^, we also investigated the possibility that the dosage compensation pathway contributes to the protogyny phenotype by knocking down the expression of *msl*-2. Finally, we evaluated the potential role of *Sxl*^27–29^.

## Results

### Environmental robustness of the protogyny phenotype in *D. melanogaster*

**Figure 1:**
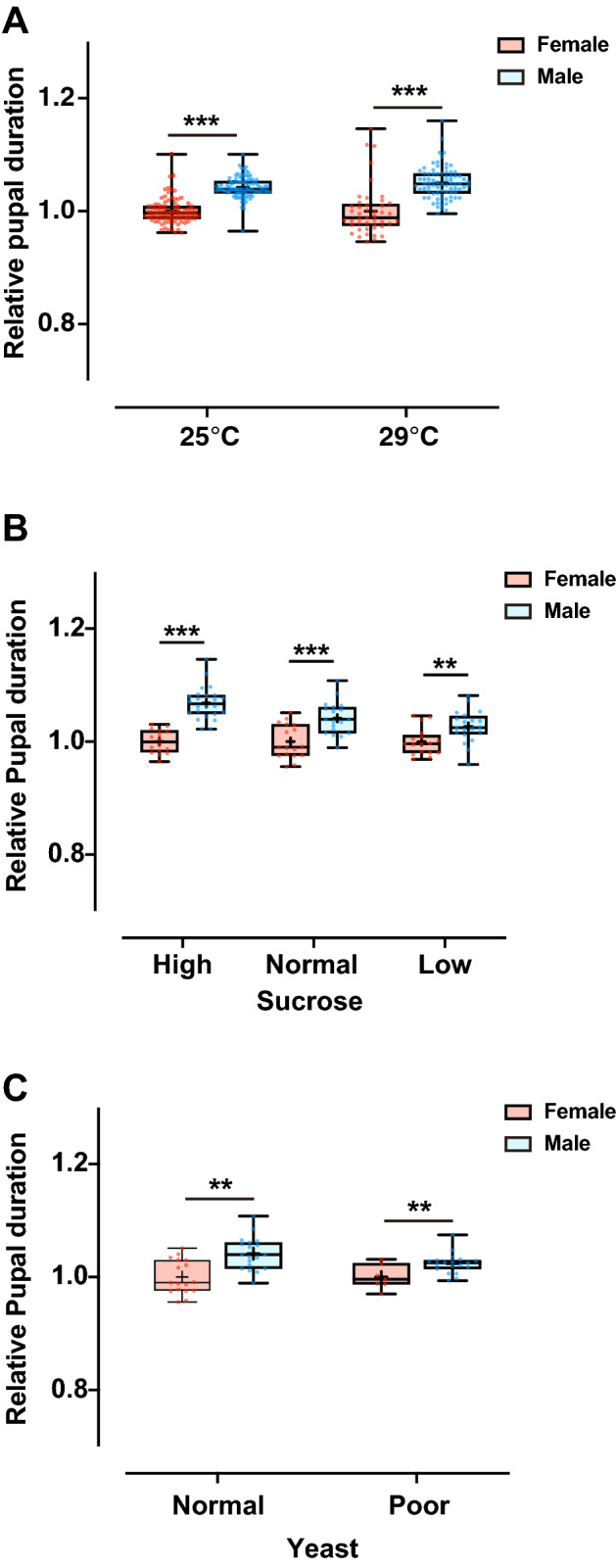
Robustness of the protogyny phenotype under varying environmental conditions. (**A**) Effect of varying temperatures (25 and 29 °C) on rearing. (**B**) Effect of sucrose concentration in the media. High, normal, and low sucrose media contained 1, 0.15, and 0.05 M sucrose, respectively, in addition to the other components of the normal medium. (**C**) Effect of yeast concentration in the media. The poor yeast medium contained one-third of the yeast concentration of standard fly medium. The number of flies analyzed is indicated in parentheses on each graph. Whiskers indicate minimum and maximum values (***p < 0.001, **p < 0.01 by Student’s unpaired *t*-test).

In *D. melanogaster*, several environmental conditions, such as temperature and nutritional conditions, influence the duration and rate of development^30–33^. Therefore, we first analyzed the environmental robustness of the protogyny phenotype in these flies. We defined the pupal duration as the duration from pupariation to eclosion and measured their timing points using our newly developed DIAMonDS tool (see “Materials and methods”)^13^. To highlight the sex-specific differences in this study, we focused on the relative pupal duration between females and males. We observed that the sex-specific difference in pupal duration was maintained at a high temperature of 29 °C (Fig. 1A, Supplementary Fig. S1A,B) as well as under varying nutritional conditions, including various concentrations of sucrose and yeast (Fig. 1B,C, Supplementary Fig. S1C,D). These results indicated that the phenotypic stability of the protogyny in *D. melanogaster* remains robust under environmental perturbation*.* Therefore, we next evaluated the molecular and genetic aspects underlying protogyny.

### Sex transformation does not affect the protogyny phenotype based on the genotype

**Figure 2:**
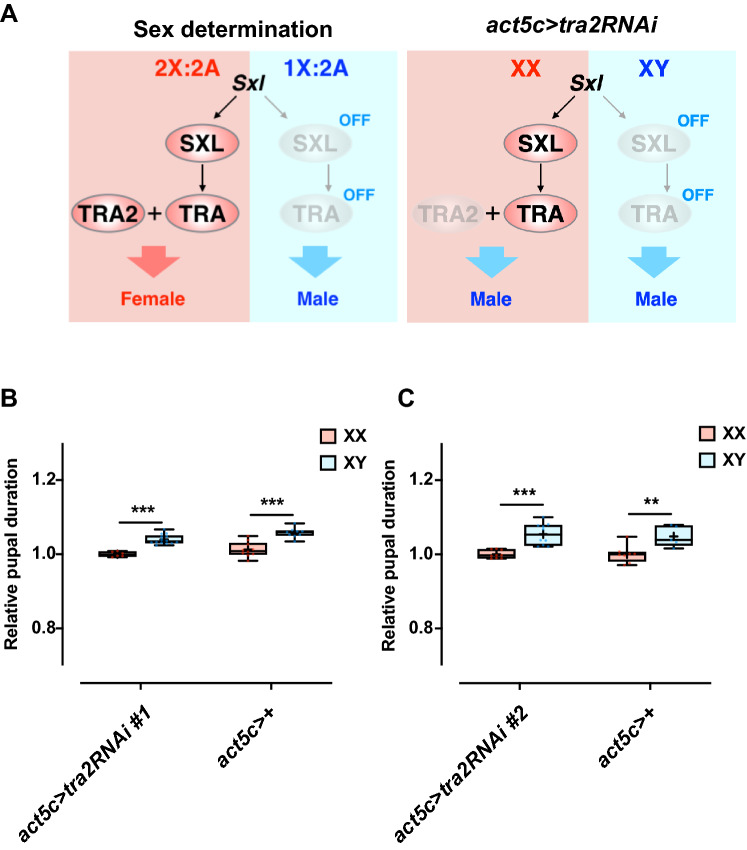
RNA interference-mediated knockdown of *tra-2* does not affect the protogyny phenotype. (**A**) Schematic presentation of the sex-determination pathway and the effect of altering *tra-2* expression. (**B**,**C**) Effect of *act5c* > *tra2* *RNAi #1* (**B**) and *act5c* > *tra2* *RNAi #2* (**C**) on the protogyny phenotype. Whiskers indicate minimum and maximum values (***p < 0.001, **p < 0.01 by Student’s unpaired *t*-test).

A previous study revealed that *tra-2* knockdown or *tra* overexpression in the whole body induces a sex transformation so that the phenotypic sex was opposite to the genotypic sex, which also altered body size (Fig. 2A)^22^. We confirmed that the phenotypic sex transformation of *D. melanogaster* can be controlled by genetic manipulation of *tra* or *tra-2* expression independent of the sexual genotype using *UAS-tra2* RNA interference (RNAi)-mediated knockdown or *UAS-traF* overexpression with ubiquitous *GAL4* drivers (Supplementary Fig. S3A). Pupal durations were then compared between siblings with XX and XY genotypes, respectively (Supplementary Fig. S2). The phenotypic transformation induced by *tra-2* knockdown or *traF* overexpression did not alter the sexual difference of pupal duration based on the chromosomal sex (Fig. 2B,C, Supplementary Fig. S3B–F). These results suggested that phenotypic sex is not critical to the protogyny phenotype, which is also independent of the *tra*/*tra-2* pathway.

### Disturbance of the dosage compensation pathway does not alter the protogyny phenotype

**Figure 3:**
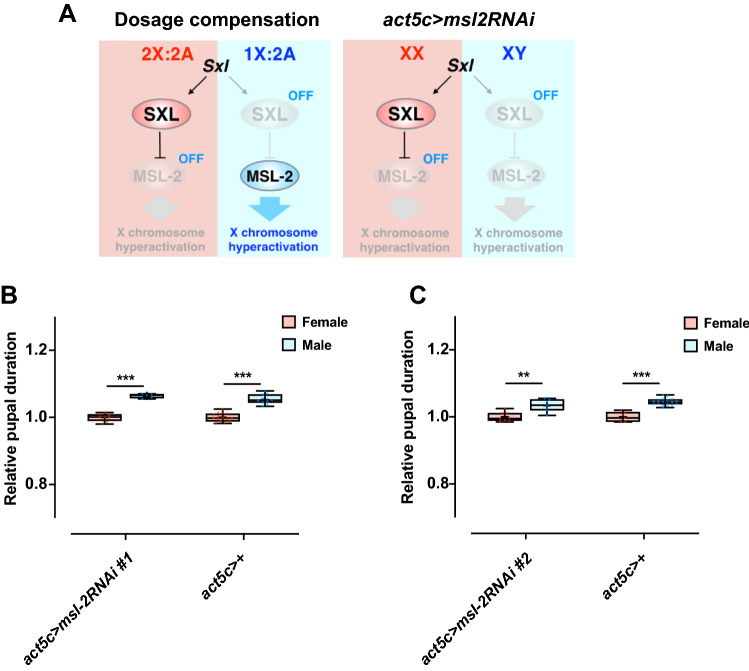
Alteration of *msl-2* expression does not affect the protogyny phenotype. (**A**) Schematic presentation of the dosage compensation pathway and effect of *msl-2* expression alteration. (**B**,**C**). Effect of *act5c* > *msl-2 RNAi #1* (**B**) and* act5c* > *msl-2 RNAi #2* (**C**) on the protogyny phenotype*.* Whiskers indicate minimum and maximum values (***p < 0.001, **p < 0.01 by Student’s unpaired *t*-test).

The dosage compensation machinery is not assembled in *Drosophila* females, because *msl-2*, a key gene involved in the assembly of the MSL complex, is repressed by Sxl (Fig. 3A)^19–21,34^. Thus, we next investigated the possible contribution of the dosage compensation pathway to the development of the protogyny phenotype. Ubiquitous knockdown of *msl-2* (Fig. 3A) successfully induced male-specific semi-lethality (Supplementary Fig. S4A), which in turn reduced the *msl-2* expression in males (Supplementary Fig. S4B). However, *msl-2* knockdown did not change the sexual difference of pupal duration, suggesting that the sex chromosome dosage compensation machinery does not commit to the protogyny phenotype (Fig. 3B,C, Supplementary Fig. S4C–F).

### Protogyny phenotype is determined in a *Sxl-*dependent manner

Although the protogyny is apparently sex-dependent, alteration of the expression of the canonical downstream components of *Sxl* did not affect the protogyny phenotype. Therefore, we next tested whether *Sxl* contributed to the protogyny phenotype.

**Figure 4:**
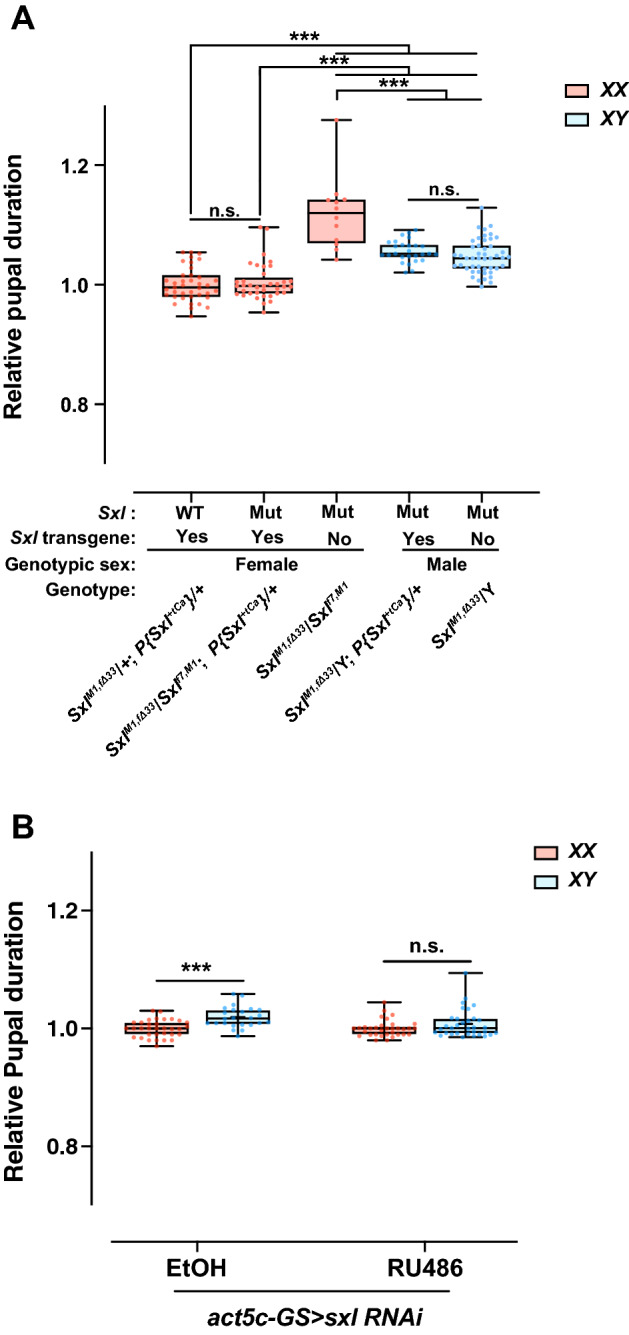
Alteration of *Sxl* expression affects the protogyny phenotype. (**A**). Effect of *Sxl* mutation on the protogyny phenotype in flies harboring or those not harboring the *Sxl* transgene. (**B**) Effect of *act5c-GS* > *Sxl* *RNAi* on the protogyny phenotype in flies grown on media with or without RU486, a glucocorticoid receptor antagonist. Whiskers indicate minimum and maximum values (***p < 0.001; n.s., no significant difference by Student’s unpaired *t*-test).

We used trans-heterozygous *Sxl*^*M1*,Δ*33*^*/Sxl*^*f7*,*M1*^ masculinized females^35–37^ and, through a crossing scheme, produced two genotypes of *Sxl*^*M1*,Δ*33*^*/Sxl*^*f7*,*M1*^ (*Sxl*^*–*^) flies with and without an extra *Sxl* transgene (Fig. 4A, Supplementary Fig. S5A). We found that *Sxl*^*–*^ females without an extra *Sxl* transgene experienced a significantly longer pupal duration than did *Sxl*^+^ females, which was reversed following introduction of an extra *Sxl* transgene, suggesting that *Sxl* may play a role in controlling sex-specific pupal duration (Fig. 4A, Supplementary Fig. S5A). The *Sxl*^*–*^ females without an extra *Sxl* transgene also exhibited a slight but significantly longer pupal duration than *Sxl*^*M1*,Δ*33*^/Y males with or without an extra *Sxl* transgene. Since the *Sxl*^*M1*,Δ*33*^*/Sxl*^*f7*,*M1*^ masculinized females have low viability (Supplementary Fig. S5B)^35^, it is possible that another mechanism regulates the longer pupal duration and delayed eclosion through the adverse effects of *Sxl* mutation in females.

To exclude the influence of *Sxl* mutation-induced adverse effects on the pupal duration, we performed RNAi-mediated *Sxl* knockdown experiments (Fig. 4B, Supplementary Fig. S5E). Since *Sxl* knockdown using *act5c-GAL4* was not successful owing to its lethal phenotype, we used a gene-switch system that induces GAL4 by administration of the glucocorticoid receptor antagonist RU486^38^. F1 larvae were derived from parents of a *UAS-Sxl* RNAi transgenic fly and an *act5c-GS-GAL4* fly reared in normal conditions, and early 3rd-instar larvae were transferred to a 96-well-microplate containing media with or without RU486. Adult F1 females eclosed from the RU486-supplemented media showed partial morphological sexual transformation, indicating that RU486-dependent *Sxl* knockdown was successful (Supplementary Fig. S6). The proportion of females was partially recovered by the stage-specific *Sxl* knockdown in comparison with that of the *Sxl*^*M1*,Δ*33*^*/Sxl*^*f7*,*M1*^ masculinized females (Supplementary Fig. S5B,F). As a control, we used *act5c-GS-GAL4/* + fly and these flies did not show any change in the protogyny phenotype under the condition with/without RU486 (Supplementary Fig. S5C,D). *act5c-GS* > *Sxl RNAi* F1 females reared in RU486-supplemented media showed longer pupal duration than the unsupplemented F1 females, and the pupal duration was similar to that of males (Supplementary Fig. S5E). Altogether, these results suggest that *Sxl* might be involved in the development of the protogyny phenotype.

## Discussion

In this study, we applied our recently developed DIAMonDS tool to explore the molecular mechanisms responsible for the slight but consistent sex difference in eclosion timing in *Drosophila* due to a difference in pupal duration.

Many morphological and physiological traits exhibit a sex difference, which may be controlled by a canonical sex-determination pathway^39^. However, the protogyny phenotype is not disturbed in genetically sex-transformed flies, established by controlling *tra* or *tra-2* expression, or by knockdown of *msl-2*. These results suggest that a morphological or physiological (dosage compensation) sex difference does not play a central role in controlling the protogyny phenotype, as manipulating these factors did not influence the length of male pupal duration. However, further genetic manipulation experiments demonstrated that the noncanonical function of *Sxl* regulates the eclosion timing and produces the protogyny phenotype in *D. melanogaster*, as females with loss-of-function mutations or knockdown of *Sxl* exhibited a pupal period of the same length as that of males (Supplementary Fig. S7).

*Sxl* expression is activated in the presence of two X chromosomes in female early embryos and is maintained via positive autoregulation^27,35,40^. Sxl also regulates splicing of its downstream components, including *tra* and *msl-2*, which play crucial roles in the sex-determination cascade and dosage compensation, respectively^15,41^. Therefore, our results suggest that the recently identified noncanonical Sxl pathways may be involved in the protogyny phenotype.

Indeed, Sxl has been suggested to interact with other targets. Nanos RNA can bind directly to Sxl in ovarian extracts, and loss-of-function studies suggested that Sxl enables the switch from germline stem cells to committed daughter cells through Nanos posttranscriptional downregulation^42^. Sxl can also bind to *Notch* to negatively control the Notch pathway^43^. Genome-wide computational screening for Sxl targets also identified an ATP-dependent RNA helicase, Rm62, as a novel potential target^44^. Rm62 was inferred to be involved in alternative splicing regulation and is required for the RNAi machinery^45,46^. A pan-neuronal RNA-binding protein of the ELAV family, found in neurons, was also shown to be downregulated by *Sxl* in female heads, independent of *tra*/*tra-2* regulation^47^. Sxl can enhance nuclear entry of the full-length Cunitus interuptus protein, suggesting a contribution to the sex difference in growth rate, although their physical interaction has not been confirmed^48^. However, there is no evidence that these noncanonical targets of Sxl directly affect eclosion timing. Therefore, further studies are required to demonstrate whether these Sxl targets, or another novel target, can contribute to the protogyny phenotype. In our study, *Sxl* mutation and whole-body *Sxl* knockdown led to delayed eclosion in females. Because loss of Sxl affects female viability, It is very difficult to completely eliminate the possibility that female sickness might induce delayed eclosion. To overcome this problem, further analysis using a novel downstream gene of *Sxl* would be necessary.

The independence of the protogyny phenotype from the canonical sex-determination pathway is very intriguing with respect to understanding the evolution of the sex difference in sexual maturation. *Sxl* does not appear to play a role in the sex determination process in most insects^37,49–51^. Several reports indicated that orthologs of *Sxl* have no sex-determinant role in non-*Drosophila* species, including in *Diptera*^50,52,53^. In Drosophilidae, ancestral *Sxl* was duplicated to *Sxl* and *sister of sex lethal* (*ssx*); the new *ssx* plays a role of ancestral *Sxl*, suggesting that *Sxl* may have evolved to function as a novel sex-determinant gene in Drosophilidae^51^. Moreover, a detailed phylogenetic study revealed that a male-specific exon, and likely embryo-specific exon, originated after the divergence between the Drosophilidae and Tephritidae families, but before the split of the *Drosophila* and *Scaptodrosophila* genera^54^. We hypothesize that the implementation of *Sxl* in the sex-determination pathway may be significantly involved in the acquisition of the protogyny phenotype in *Drosophila*. Therefore, we expect that identification of the target of the noncanonical *Sxl* sex-specific regulation for the protogyny phenotype may help to promote a better understanding of the evolutionary aspects of protogyny.

## Methods

### *Drosophila* stocks

All flies were maintained at 25 °C on standard laboratory medium as described previously^55^. The following stocks were obtained from the Bloomington Drosophila stock center (BDSC): *w*^*1118*^ (wild-type; BDSC 5905), *act5c-GAL4* (BDSC 3954)*, da-GAL4* (BDSC8641)*, elav-GAL4* (BDSC 458)*, elav-GAL4; UAS-dcr-2* (BDSC 25750)*, P{CaryP} attP2* (BDSC 36303)*, UAS-tra2 RNAi #1* (BDSC 56912)*, UAS-tra2 RNAi #2* (BDSC 28018), *UAS-traF* (BDSC 4590)*, UAS-msl-2 RNAi #1* (BDSC 31627)*, UAS-msl-2 RNAi #2* (BDSC 35390)*, UAS-Sxl RNAi #1* (BDSC 34393)*, UAS-Sxl RNAi #2* (BDSC 38195), *Sxl*^*f7,M1*^*; P{Sxl.* + *tCa}9A/* + (BDSC 58486)*,* and *Sxl*^*M1,fΔ33*^*/Binsinscy* (BDSC 58487). Three gene-switch *Gal4* driver lines—*act5c-GS-GAL4, S106-GS-GAL4,* and *5961-GS-GAL4*—were kindly gifted by Dr. Akagi^56^.

### Measurement of pupal duration

We used our recently developed DIAMonDS tool to measure pupal duration at the individual level. The wandering 3rd-instar larvae were collected from rearing vials, and a single larva was placed in the well of a 96-well microplate with standard medium. The plate was then placed on a flatbed CCD scanner to obtain time-lapse images until all flies were eclosed. The time-lapse image dataset was then analyzed using Sapphire software as described previously^13^.

To compare the effect of *Sxl* mutation on pupal duration, *Sxl*^*f7,M1*^*; P{Sxl.* + *tCa}9A/* + females were crossed with *w*^*1118*^/*Y* males. The F1 progeny *Sxl*^*f7,M1*^*/Y; P{Sxl.* + *tCa}9A/* + males were then crossed with *Sxl*^*M1,fΔ33*^*/Binsinscy* females. Each genotype of the F2 flies was then assessed for pupal duration using DIAMonDS. To induce the gene-switch *Gal4* driver, RU486 (Mifepristone; Sigma, St. Louis, MO, USA) reagent was dissolved in ethanol and added to the medium at a final concentration of 100 µg/mL.

To detect the sex genotype of the flies, genomic DNA was extracted from single adults by homogenization in 50 μL of squishing buffer (10 mM Tris–HCl [pH 8.2], 1 mM EDTA, 25 mM NaCl, and 200 μg/mL proteinase K) and incubated at room temperature for 20 min, followed by inactivation at 95 °C for 5 min. The extracted genomic DNA was subjected to polymerase chain reaction analysis using a WD repeat-containing protein on the Y chromosome (*WDY*)- and *Rp49*-specific primer mix by ampliTaq Gold 360 master mix (Applied Biosystems, Waltham, MA, USA), following which the amplified DNA fragments were separated by 2% agarose gel electrophoresis (Supplementary Fig. S1).

### Reverse transcription-quantitative polymerase chain reaction (RT-qPCR)

Total RNA was extracted from the whole adult body (for measuring *msl-2* expression knocked down by *act5c-GAL4*) and dissected larval central nervous system (for measuring *Sxl* expression knocked down by *elav-GAL4*) using Isogen II (Nippon Gene, Tokyo, Japan). RT-qPCR was performed using a One Step SYBR PrimeScript PLUS RT-PCR kit (Takara Bio, Shiga, Japan) and Applied Biosystems ABI Prism 7000 Sequence Detection System. All mRNA levels were normalized to those of *rp49*. We used the following primers for RT-qPCR (5′–3′): *Sxl*, forward primer (5ʹ-CCAATCTGCCGCGTACCATA-3ʹ), reverse primer (5ʹ-AATGGAACCGTACTTGCCGA-3ʹ); *msl-2*, forward primer (5ʹ-CACTGCGGTCACACTGGCTTCGCTCAG-3ʹ), reverse primer (5ʹ-CTCCTGGGCTAGTTACCTGCAATTCCTC-3ʹ); and *rp49*, forward primer (5ʹ-GATGACCATCCGCCCAGCATAC-3ʹ), reverse primer (5ʹ-AGTAAACGCGGTTCTGCATGAGC-3ʹ).

### Statistical analysis

All data were analyzed and graphs were plotted using Prism 8 (GraphPad Software, San Diego, CA, USA). Data are presented as the mean ± standard deviation. Student’s unpaired two-tailed *t*-test was performed to compare differences between two groups in each experiment, and Dunnett’s one-way analysis of variance was used for multiple comparisons; *p* < 0.05 was considered to indicate a statistically significant difference.

## Supplementary Information

Supplementary Figure S1.

Supplementary Figure S2.

Supplementary Figure S3.

Supplementary Figure S4.

Supplementary Figure S5.

Supplementary Figure S6.

Supplementary Figure S7.

Supplementary Table 1.

## Abbreviations

CCD: Charge-coupled device
BDSC: Bloomington *Drosophila* stock center
dsx: Doublesex
DIAMonDS: *Drosophila* Individual Activity Monitoring and Detection System
*msl-2*: *Male-specific lethal-2*
RT-qPCR: Reverse transcription-quantitative polymerase chain reaction
RNAi: RNA interference
*Sxl*: *Sex lethal*
*ssx*: *Sister of sex lethal*
*tra*: *Transformer*
*WDY*: *WD repeat-containing protein on the Y chromosome*
